# Role of the mitral valve in left ventricular assist device pathophysiology

**DOI:** 10.3389/fcvm.2022.1018295

**Published:** 2022-10-28

**Authors:** Pierre-Emmanuel Noly, Neal Duggal, Mulan Jiang, David Nordsletten, Mia Bonini, Ienglam Lei, Ashraf Abou El Ela, Jonathan W. Haft, Francis D. Pagani, Thomas M. Cascino, Paul C. Tang

**Affiliations:** ^1^Department of Cardiac Surgery, Montreal Heart Institute, Université de Montréal, Montréal, QC, Canada; ^2^Michigan Medicine, University of Michigan, Ann Arbor, MI, United States; ^3^Massachusetts Institute of Technology, Cambridge, MA, United States; ^4^Department of Biomedical Engineering and Cardiac Surgery, Frankel Cardiovascular Center, University of Michigan, Ann Arbor, MI, United States; ^5^Department of Cardiac Surgery, School of Medicine, University of Michigan, Ann Arbor, MI, United States; ^6^Division of Cardiovascular Medicine, Department of Internal Medicine, School of Medicine, Michigan Medicine, University of Michigan, Ann Arbor, MI, United States

**Keywords:** advanced heart failure, functional mitral regurgitation, mitral valve, left ventricular assist device, transcatheter edge-to-edge therapy, right heart failure, aortic valve, tricuspid valve

## Abstract

Functional mitral regurgitation (MR) in the setting of heart failure results from progressive dilatation of the left ventricle (LV) and mitral annulus. This leads to leaflet tethering with posterior displacement. Contrary to common assumptions, MR often does not resolve with LVAD decompression of the LV alone. The negative impact of significant (moderate-severe) mitral regurgitation in the LVAD setting is becoming better recognized in terms of its harmful effect on right heart function, pulmonary vascular resistance and hospital readmissions. However, controversies remain regarding the threshold for intervention and management. At present, there are no consensus indications for the repair of significant mitral regurgitation at the time of LVAD implantation due to the conflicting data regarding potential adverse effects of MR on clinical outcomes. In this review, we summarize the current understanding of MR pathophysiology in patients supported with LVAD and potential future management strategies.

## Introduction

Functional mitral regurgitation (MR) occurring in end-stage heart failure results from progressive dilatation of the left ventricle (LV) and mitral annulus driven by progressive left ventricular dysfunction. LV dilation leads to leaflet tethering with posterior displacement (1) accompanied by change of LV geometry from an elliptical to a spherical shape (2, 3). Outward papillary muscle displacement also contributes to mitral leaflet tethering (4, 5). This pathological cardiac remodeling process can occur in both ischemic and non-ischemic cardiomyopathies (6). It is more recently recognized that “atrial functional mitral regurgitation” plays an important role for MR pathogenesis in heart failure. This describes structural left atrial remodeling and dilatation which is commonly associated with atrial fibrillation. This atrial enlargement occurs and contributes to the normal elliptical and saddle shaped mitral annulus becoming rounder and flatter (7). Atrial functional MR is also characterized by isolated mitral annular dilatation, inadequate leaflet growth/adaptation as well as impaired atrial and annular contractile dynamics (8). Interestingly, the association of left atrial dilatation with functional MR was initially described in patients with atrial fibrillation (9).

The occurrence of functional MR in the heart failure setting is common. There is a 44.5% prevalence of moderate-severe MR in patients with acute heart failure with reduced ejection fraction (10). As expected, this correlated closely with the 39–43% incidence of preoperative significant MR in patients undergoing left ventricular assist device (LVAD) implantation (11, 12). The large majority (∼94%) of MR in LVAD patients are a result of restricted leaflet motion during systole from tethering (type IIIb) along with components of reduced leaflet motion from thickening and calcification (type IIIa) and annular dilatation (type I) (13). It should be noted that there are significant challenges in evaluating the burden of preoperative and residual MR given its underlying dynamic nature where MR severity is modulated by conditions such as pump speed, afterload and volume status. Aggressive medical optimization to promote euvolemia, blood pressure control and speed adjustments to promote optimal LVAD support should be carried out prior to assessing MR severity with subsequent interventions.

Despite common assumptions, MR often does not resolve with LVAD support alone. In patients with preoperative moderate-severe MR, up to 34% had persistent significant MR on follow-up. This is more likely with greater posterior displacement of the coaptation point (1). Therefore, a significant number of patients have moderate to severe MR following LVAD implant. This proportion is particularly high in those with severe MR preoperatively. Despite recent reports citing the negative impact of persistent MR after LVAD implantation, reaching a consensus on interventions for moderate-severe MR remains controversial (14).

This impact of significant (moderate-severe) mitral regurgitation (SMR) in the LVAD setting is gradually being recognized. However, there remain important controversies regarding its implications as well as management. There are a number of explanations for these. Several studies have found that preoperative SMR does not impact post-LVAD surgical outcomes or survival, but many studies did not specifically examine those patients with persistent SMR following LVAD implantation (1, 12, 15, 16). Intuitively, it would be the persistence of post-LVAD SMR that are more likely impact LVAD outcomes over time, not preoperative MR severity *per se*. At present, there are no consensus indications for repair of SMR at the time of LVAD implantation due to the conflicting data regarding its potential adverse effects on clinical outcomes (14).

## Mitral regurgitation and its impact on left ventricular assist device outcomes

Residual SMR after LVAD can increase pulmonary vascular resistance, negatively impact right ventricular function, promote right ventricular failure, increase hospital readmissions, and likely reduce survival in settings such as destination therapy (17, 18). While it is recognized that LVAD therapy will improve pulmonary hypertension over time (19), Kassis et al. reported that the presence of residual SMR after LVAD implantation are more likely to have persistent pulmonary hypertension, and increased mortality (20). Importantly, Taghavi et al. observed in patients with significant preoperative MR that concomitant mitral surgery with LVAD implant led to a greater reduction in mean pulmonary artery pressures and pulmonary vascular resistance (PVR) compared to those without concomitant mitral intervention (16). Computational modeling showed that at LVAD speeds where AV opening occurs, moderate-severe MR was associated with significant increases in pulmonary artery and left atrial pressures (21). Elevations in pulmonary vascular pressures and resistance will also negatively impact heart transplant candidacy (16). The impact of residual significant MR on right ventricular failure (RVF) will be discussed in the section below.

Given the purported negative impact of residual SMR on right heart physiology, a number of studies investigated its impact on defined clinical outcomes. However, the results of these studies have yielded varying results. One group of studies found that moderate-severe MR did not adversely impact LVAD outcomes. Kawabori et al. retrospectively studied patients with preoperative severe MR (*n* = 108) and found that those who underwent mitral valve (MV, *n* = 26) repair did not influence survival, postoperative right heart failure, or readmission (22). Studies by Stulak et al. concluded that preoperative significant MR (*n* = 189, 39%) did not adversely impact outcomes. In fact, the presence of larger preoperative end-diastolic dimensions was actually marker by improved survival after LVAD implant, particularly in those with centrifugal devices (12).

Conversely, other studies found mitral regurgitation had significant effects on quality of life, hospital readmissions and survival. Robertson et al. conducted an INTERMACS registry study (*n* = 4,930) for patients with preoperative significant MR and found that mitral intervention (*n* = 263) only demonstrated a trend toward improved survival (*P* = 0.089) in those with destination therapy indications (17). However, when examining the entire INTERMACS population, MV repair/replacement did not impact 2-year survival compared to those who did not. Despite this, patients who underwent MV procedures had a lower rate of readmission and a better quality of life (17). The clinical impact of significant residual MR translates most consistently with its influence on increased readmission rates. This is most likely the result of a higher incidence of RVF in those with residual SMR (13, 23).

## Residual mitral regurgitation and right ventricular failure

Postoperative RVF occurs in 29.8 to 38.5% following LVAD implant and is an important challenge to successful durable LVAD therapy (24, 25). RVF is associated with serious complications such as postoperative bleeding, multi-organ failure, and thromboembolic issues (26). Severe RVF requiring right ventricular assist device (RVAD) support increases hospital mortality. Despite eventual successful RVAD weaning, these patients still often experience an increased incidence of future heart failure (27). Several right ventricular failure (RVF) risk prediction models have been developed for use in patient selection for LVAD therapy (28). Unfortunately, the accuracy of these models have been modest in predicting postoperative RVF. Multiple well-recognized RVF prediction models have only a 60% positive predictive value at best (29). This is likely because existing models only reflect an incomplete portion of a myriad risk factors that all contribute to RVF in the LVAD setting. These unaccounted for risk factors may include intrinsic myocardial biology, systemic inflammatory milieu and/or associated valvular pathologies. For the purpose of this review, the discussion will be focused on mitral regurgitation as a contributor to RVF.

We found that patients with larger preoperative cardiac dimensions had a higher incidence of significant residual MR. These patients were two times more likely to have severe RV dysfunction and over three times the rate of manifesting the clinical symptoms of late RV failure. Late RV failure also highly correlated with lower survival (*P* = 0.006) (30). Kassis et al. reported similar findings where postoperative LVEDD and RV dimensions was larger in patients with significant residual MR and this was associated with worse RV function by quantitative parameters (20). This is likely a result of consistently elevated afterload demands on the RV resulting from increased pulmonary vascular resistance which contributes to RV failure over a prolonged period (30). Kapelios et al. also reported the entity of late-onset RVF during LVAD support where RVF can occur many months to years from device implantation. This was show to be associated with poorer outcomes such as mortality and survival to heart transplantation (31).

**FIGURE 1:**
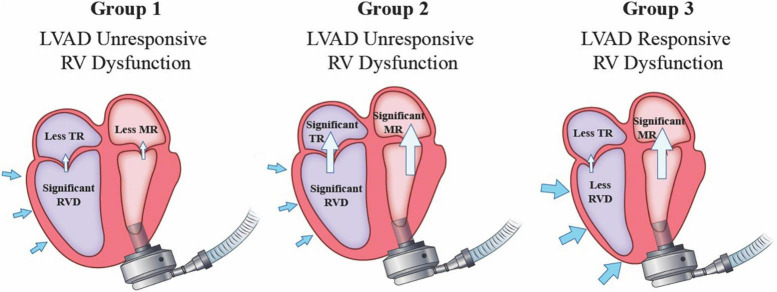
Post-LVAD implantation echocardiographic findings on atrioventricular valve competency and underlying right heart function determines the risk of postoperative right ventricular failure.

While preoperative MR severity is important for subsequent decision making on anticipated need for mitral intervention, it is actually the postoperative residual MR that understandably determines eventual impact. We examined 159 patients with pre-LVAD severe MR and determined the impact of MR resolution after LVAD. Our studies show that persistent post-LVAD SMR in combination with moderate-severe RV dysfunction had very poor outcomes. We documented a high rate of stroke (30.2%), RVF (20.9%), hemolysis (39.5%) and RVAD use (18.6%) in this group which likely contributed to a lower survival in this population (32). However, in patients with post-LVAD significant RV dysfunction but resolution of MR, there was a relatively low incidence of RVF (9%) and RVAD use (7.5%) (32). On the other hand, in patients with more preserved RV function, the presence of SMR post-LVAD was well-tolerated with a very low incidence of RVF (2%) (32). Thus, in patients presenting with moderate-severe MR for LVAD implantation, a favorable outcome is associated with MR that improves to mild or less in severity and/or the RV function is relatively normal after continuous flow LVAD implantation (Figure 1; 32).

## Resolution of mitral regurgitation in the left ventricular assist device setting

There is controversy regarding the indications for surgical intervention for significant mitral regurgitation (MR) associated with continuous flow left ventricular assist device (cfLVAD) therapy (12). Concomitant mitral surgery during LVAD implantation is performed only in about 5% of patients with preoperative significant MR (17). The International Society of Heart Lung Transplantation (ISHLT) guidelines do not provide a recommendation for concomitant mitral surgery at the time of LVAD implantation (14, 33). This “no-intervention” approach is based on the expectation that LVAD support itself will decrease the ventricular dimensions to resolve MR (34). However, there may be non-responders for MR improvement after LVAD implant. Increasing LVAD speed alone to resolve MR may conflict with competing goals of optimizing right ventricular function, promoting aortic valve opening and avoidance of suction events.

Pawale et al. reported that MV repair can be done safely with excellent outcomes in reducing MR during cfLVAD implant (35). However, Tanaka et al. reported that in patients with significant preoperative MR who spontaneously corrected their MR without a MV procedure after cfLVAD implant, recurrent MR occurred in 23–25% during mid-term follow up at just over 1 year. Additional reports of recurrent MR were also observed by other investigators (1, 36, 37). It is should be highlighted that mitral valve repair may lead to greater reductions in PVR which reduces right ventricular work and may also lower the incidence of heart failure related readmissions (38). While severe MR can predict postoperative RVF and RVAD use in the immediate postoperative setting, persistent MR also likely has important implications for long term outcomes (39).

The rate of MR resolution following LVAD implantation likely varies according to the severity of pre-LVAD MR. Studies commonly grouped together pre-LVAD moderate and severe MR when assessing MR resolution (1, 12, 40). Morgan et al. reported that while 76% of patients had either moderate or severe MR pre-LVAD, this declined to 8% at 6 months post-LVAD following LVAD implantation (40). In the Momentum trial, Kanwar et al. studied 403 patients undergoing LVAD implant with preoperative moderate or severe MR. At 1 month, only 6.2% of patients with HM3 and 14.3% with HMII had significant residual MR (11). Further analysis showed that patients are more likely to have significant residual MR if they have MR classified as severe, larger preoperative left ventricular dimension and use of a HeartMate II device (11). Therefore, patients with pre-LVAD severe MR are likely to be an important target population when designing interventions that address residual SMR. When we focused on patients with pre-LVAD severe MR, we found that LVAD support only reduced MR to mild or less in 69.3% of patients. After LVAD implantation in this population, MR remained severe in 10.7% and moderate in 27.0% (32). By comparison, only 16% of those with pre-LVAD moderate MR had significant residual MR after LVAD implantation (41). Posterior displacement of the coaptation point was also an important predictor of MR non-resolution (1). This suggests that while a significant majority resolved SMR with LVAD support alone, those meeting criteria for pre-LVAD severe MR are much less likely to do so.

**FIGURE 2:**
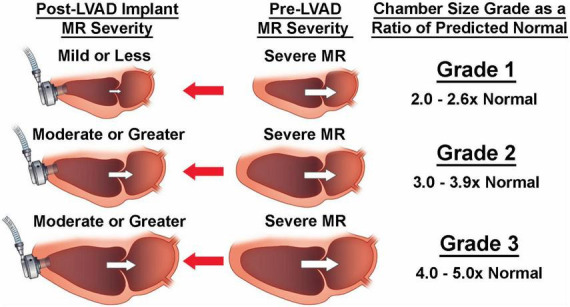
Despite decompression by left ventricular assist device (LVAD) implantation, larger pre-LVAD left heart dimensions (grades 2 and 3) are less likely to down size sufficiently to allow mitral leaflet coaptation due to severe tethering.

Building upon findings by previous groups on predictor of residual SMR, we employed a non-hypothesis driven statistical phenotyping of cardiac chamber dimensions. This revealed the correlations between pre-cfLVAD chamber size to MV tenting, early and late post-cfLVAD MR resolution and the occurrence of RV failure (30). Interestingly, LV and left atrial (LA) sizes greater than 3 times the normalized dimensions had twice the risk of having residual SMR at last follow up compared to those less than 3 times the normal size (50–55% vs. 25% respectively). Increased LA, LV, and mitral annular sizes were all significantly associated with post-cfLVAD MR severity. However, LA dimensions had the strongest correlation which is consistent with the now recognized contribution of LA dilatation to functional MR. Larger LA are more likely to have elevated atrial pressures, mitral annular dilatation, LA fibrosis, impaired atrial systole/diastolic function (8). Indeed, very large hearts (Figure 2; 30) had the greatest LA volumes despite LVAD decompression of the ventricle and also had the largest incidence of residual SMR (55.6%) (30).

It is important to note while LVAD decompression greatly reduced cardiac volume, mitral annular dimensions, and leaflet tenting, this may did not correlate with leaflet coaptation nor MR resolution in patients with very large hearts (30). This is congruent with Kitada et al.’s findings where preoperative posterior displacement of the mitral leaflet coaptation point was a predictor for significant residual MR at 1 week following LVAD implant (1). Thus, in the setting of extreme baseline leaflet tethering associated with a very dilated LV, mitral leaflet coaptation may not be achieved despite the maximum degree of LVAD decompression. LVADs decompression of the LV is limited by its negative impact on right ventricular (RV) function as well as competing goals of promoting aortic valve (AV) opening and native LV ejection.

While 37.7% of patients with pre-LVAD severe MR had residual SMR, only 16% of those with moderate MR pre-LVAD had residual SMR (41). Therefore, a great majority (84%) of patients with pre-LVAD moderate MR had improvement to mild or less following device implant (41). Indeed, indications for surgical intervention for moderate MR in other non-heart failure settings (e.g., coronary bypass grafting) have been more controversial than the general consensus to correct severe MR (42–44). Given data suggesting a different rate of preoperative moderate vs. severe MR resolution after LVAD, there are unique considerations when faced with moderate MR in a LVAD candidate. Importantly, patients with residual SMR had greater preoperative LVEDD and LVESD and this population may be further defined in future studies for prediction of MR resolution. This also supports findings in the Momentum 3 trial where greater LV dimensions were predictive for residual significant MR in the combined moderate and severe MR groups (45). It is possible that moderate MR patients with larger LV dimensions may be identified for MV intervention. However, the patient population selected for mitral intervention during LVAD implantation needs to be accurately selected to avoid unnecessary procedures and prolonged cardiopulmonary bypass times in these high-risk patients.

## Interaction of residual mitral regurgitation with aortic and tricuspid valve pathologies

### Mitral regurgitation and the tricuspid valve

Patients with heart failure often have associated single or multi-valvular pathologies (46). However, most studies have focused on single valvular lesions when assessing their impact on postoperative outcomes such as impact on right ventricular (RV) function. The complexity of RVF pathogenesis post-cfLVAD means that it is unlikely to be fully accounted for by a single valvular lesion. Concomitant tricuspid regurgitation is highly prevalent in those presenting with mitral valve pathologies (47, 48), and is also frequently observed in patients undergoing surgical or transcatheter aortic valve replacement (TAVR) for aortic stenosis (49–51). For example, the mitral and tricuspid valves exist in series with the tricuspid valve being upstream in location and subject to forces exerted in a retrograde direction. Indeed, severe regurgitation of both mitral and tricuspid valve in the setting of biventricular failure had the highest incidence of post-LVAD RVF (20.3%) and RVAD use (17.2%) (39). The presence of significant tricuspid regurgitation may reflect several contributing mechanisms to RV dysfunction. Increased PVR from pre-LVAD persistent MR may lead to long standing RV dysfunction with remodeling and enlargement of the tricuspid annulus. This is a good indicator and likely contributes to predictably poor RV function after LVAD implant. Indeed, Accordingly, in the absence of associated moderate-severe TR in LVAD patients with severe MR, this phenotype is associated with a low incidence of RVF (5.5%) and RVAD utilization (4.5%) (39). In this setting associated pre-LVAD right ventricular dysfunction will likely be improved with LVAD support and diuresis since the tricuspid annulus is no chronically dilated from long standing RV dysfunction.

The presence of both severe TR and RV dysfunction is also highly associated with RVF with an Odds Ratio (OR) of 3.22. Echocardiographic evidence of moderate-severe RV dysfunction with moderate or less TR is a much weaker RVF predictor with an OR 1.78 (*P* = 0.009). This association with RVF is further strengthened if the patient also has severe MR along with significant RV dysfunction (39). A plausible explanation is that if severe TR persists despite diuresis and medical optimization, then this likely indicates long standing tricuspid structural remodeling with annular enlargement associated with chronic RV dysfunction as distinct from acute volume overload (39). Whether the finding of severe TR is a marker of significant underlying RV dysfunction as opposed to having an independent role in reducing RV forward flow remains less well-defined. Nevertheless, the implication is that TV repair for severe TR may not significantly improve RV function if residual SMR is present after LVAD implantation. Residual SMR will likely impair RV performance by increasing pulmonary artery pressures and afterload.

Indeed, we demonstrated that greater postoperative MR severity correlated independently with RVF (OR = 1.6) and RVAD use (OR = 1.6). We also excluded patients who underwent concomitant TV surgery and showed a strong positive correlation between the degree of post-cfLVAD MR and TR severity which suggests that residual MR imposes significant afterload on the right heart (32). It is likely that the population with significant residual MR coupled with moderate-severe RV dysfunction are most likely to benefit from restoring MV competency.

Currently, our practice is that severe TR especially in patients with a dilated tricuspid annulus are addressed with TV repair. The decision for TV repair is also determined by surgeon preference, and moderate TR is increasingly intervened upon over time. Until recently, MR was typically not repaired even if severe. Moderate-severe AI is uniformly addressed intraoperatively but lesser degrees of AI have also been addressed by our group more recently as per surgeon preference.

### Mitral regurgitation and the aortic valve

The combined effects of aortic and mitral regurgitation in the LVAD setting are not well-studied. However, studies of double left sided valve regurgitations in the non-LVAD literature have documented severe volume and pressure overload which is poorly tolerated as expected. LV remodeling in this setting is characterized by severe dilatation combined with an eccentric hypertrophic remodeling pattern (that is lower wall thickness to cavity ratio). Importantly, the presence of premature mitral valve closure which limits the flow reversal into the left atrium in severe aortic regurgitation contributes to poor clinical outcomes (52). Symptomatic patients with this pattern of valve lesions have worse LV function than those with isolated aortic or mitral regurgitation (53, 54). In the LVAD setting it would be expected that regurgitant volumes will be larger than the non-LVAD setting given mechanically driven continuous flow which is rapidly re-circulated. Native ejection if any, would also be reduced given greatly impaired forward flow.

Cowger et al. described progressive aortic insufficiency (AI) in LVAD patients contributing to worsening MR and this adversely impacted RV function (55). Indeed, in patients with pre-LVAD significant (moderate-severe) aortic insufficiency (AI) there was already a very high incidence of moderate-severe RV dysfunction (62.5%) and severe MR (38.9%) (39). While several studies have focused on new-onset AI after a lengthy duration of LVAD support (56), the implications of preoperative isolated AI are less clear. Interestingly, we showed that preoperative RV dysfunction associated with concomitant significant AI rarely results in severe RV dysfunction after LVAD implant especially when it is not accompanied by mod-severe TR (39). Since temporary mechanical circulatory support is generally contraindicated in the presence of severe AI, this may have contribute to timely LVAD implantation with AV intervention in this group. Furthermore, concomitant significant MR and AI can present with early symptoms resulting in prompt intervention. This may reduce the duration of exposure of the RV to elevated left sided pressures.

## Approach to presence of MitraClip during left ventricular assist device implant

The MitraClip is increasingly used to address functional mitral regurgitation through transcatheter coaptation of mitral leaflets (57, 58). Although improvement of clinical symptoms and better exercise tolerance has been reported (57, 59, 60), controversies exist as to whether it translates into reduced heart failure admissions or improved survival (58, 59, 61–63). Regardless, a portion of patients treated with MitraClip do subsequently undergo LVAD implantation. The average mitral orifice area reduction from MitraClip is about 40–50% (64). It is important to carefully echocardiographically assess the mitral valve pre-LVAD, intraoperatively and post-LVAD. When LVAD candidates with MitraClip are assessed, the implanting team should ascertain how many Clips were placed as greater than 3 clips is likely to increase transmitral gradients after the low flow state is corrected by the LVAD (65). In practice, any clips causing more than mild stenosis pre-LVAD will likely need to be addressed (65). In the MitraBridge study, where 119 patients on the heart transplant list was treated with MitraClip, about 12.5% of patients had 3 or more Clips (66).

It is important to assess whether significant MR is present following MitraClip placement. This may indicate Clip dehiscence, single leaflet device implant, mitral leaflet injury (e.g., perforation) or thrombus formation on the Clip that may need to be addressed intraoperatively (67). In the absence of mitral stenosis or MitraClip specific issues, the clips can generally be left in place as it will help mitigate against significant residual MR. It is critical to re-assess intraoperative mitral gradients following full LVAD support to rule out mitral stenosis in the presence of normal flow volumes across the mitral annulus.

If mitral stenosis or MitraClip complications are present however, the surgical team needs to assess mitral apparatus integrity, mitral annulus and orifice size, magnitude of the transmitral gradient, and overall left heart dimensions. Indeed, larger left atrial and ventricular volumes are associated with an increased incidence of significant residual MR (30). If mitral stenosis is deemed present or likely, an attempt can be made to remove excess MitraClips if the mitral valve apparatus is not compromised. However, scarring around the device can be a barrier to effective removal. If all MitraClips are removed and cardiac dimensions are high, then an annuloplasty ring and/or an Alfieri central coaptation stitch should be considered to minimize the negative impact of significant residual MR.

## Concomitant mitral repair with annuloplasty

Persistent MR also works against the LV pressure that can be produced by the ventricular myocardium to augment cardiac output as well as negatively impacting the ability to open the aortic valve consistently (68). Importantly, the use of cfLVAD support to reduce chamber and annular size needed to be balanced with the risk of septal shift resulting in worsened RV function (21). While it has been suggested that MR may not be relevant when considering the average lifespan of 4 years for destination therapy patients (69), this rationale may become less relevant as LVAD technology increases in effectiveness, reliability and longevity. As suggested by Taghavi et al. and Tanaka et al., surgical correction of MR is an effective and reliable intervention for those who are likely to remain with significant MR following cfLVAD implant and can improve LVAD outcomes (36).

We find that a full annuloplasty ring that is 30 mm or greater will not usually cause stenosis in the LVAD setting. However, if the mitral valve apparatus is irrevocably damaged, we recommend mitral valve replacement (bioprosthesis) with chordal sparing. We prefer a transseptal approach to the mitral valve in this setting as it allows access to the tricuspid valve if intervention is planned and provides excellent hemostasis. The left atrium is often very large in patients with severe pre-LVAD MR and affords an excellent view of the mitral valve. Mitral intervention can often be performed without aortic cross-clamping in the presence of a competent aortic valve. To minimize the risk of air embolism in this setting, we vent the heart through the left atrium (*via* right superior pulmonary vein), left ventricular apex and ascending aorta. The iatrogenic atrial septal defect from the 24 French MitraClip catheter often resolves in 73% of patients by 1 year (70). If present however, we do close this to avoid systemic thromboembolic events, worsening of right ventricular function by left to right shunting or arterial desaturations from right to left shunting (70). More technical details on mitral surgery during LVAD implantation will be discussed separately in this topic series.

There have been concerns that concomitant MV surgery may increase the surgical risk due to increased cardiopulmonary bypass times and needing to cross-clamp the aorta in select settings such as aortic insufficiency. Indeed, longer cardiopulmonary bypass duration during LVAD surgery contributes to per-operative vasoplegia (71). We suggest that although aortic cross-clamping is at times necessary, concomitant MV intervention can often be done without cardioplegic arrest thus avoiding ischemic injury to the right heart (36). Furthermore, we target patients with larger cardiac sizes that are less likely to resolve MR with LVAD alone. These candidates often have very large atria and ventricles which afford excellent visualization of the mitral valve for expeditious surgical intervention.

## Intervention for atrial fibrillation during left ventricular assist device implant

Co-existing atrial fibrillation and heart failure with reduced ejection fraction (HFrEF) commonly occur. Importantly, this combination of pathologies is associated with an increased risk of all-cause mortality and morbidity compared to either condition alone. Presence of both atrial fibrillation and HFrEF is associated with a higher risk of hospitalization, stroke, myocardial infarction, renal failure and death than in patients with either condition in isolation (72, 73). About 50% of patients presenting for mitral valve surgery have atrial fibrillation (74, 75). In comparison, a history of atrial fibrillation is present in 21–54% of LVAD patients (72, 76–79). Atrial fibrillation associated with LVAD therapy increases thromboembolic events such disabling strokes as well as pump thrombosis (72, 76, 80–82). Furthermore, atrial fibrillation in the LVAD setting is associated with right ventricular failure and elevated right atrial pressures (83). Increased ventricular rate from atrial fibrillation can contribute to right ventricular failure (84–86). Left atrial appendage ligation is associated with reduced risk of stroke in patients with atrial fibrillation (87, 88). Left atrial appendage ligation at the time of LVAD implantation has been performed either routinely (80) or only in the setting of atrial arrhythmias (81). This has been shown to decrease the rate of disabling stroke in LVAD patients (80). Our group currently performs left atrial appendage ligation in patients with atrial arrhythmias. This is achieved using the commercially available AtriClip or with an excise-and-sew technique with 4-0 or 5-0 prolene in 2 layers. It should be noted that the AtriClip will need to be excised if subsequent heart transplantation is performed but this is can usually be accomplished without great inconvenience.

## Transcriptomic biology of mitral regurgitation in end-stage heart failure

**FIGURE 3:**
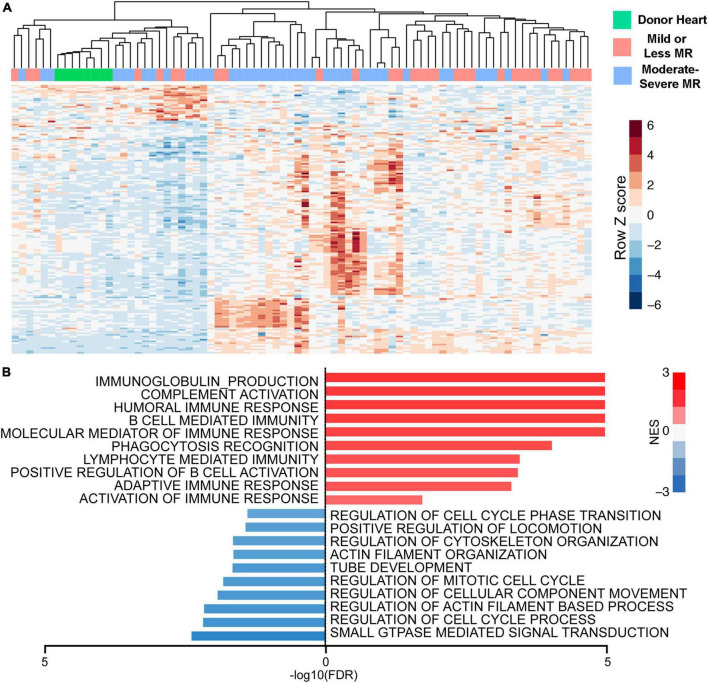
Greater mitral regurgitation (MR) severity is associated with heightened expression of immune response genes and down-regulation of genes associated with cellular proliferation and structure. **(A)** Heatmap of subtype specific differentially expressed genes (DEGs). **(B)** Gene set enrichment analysis using moderate-severe MR-specific regulated genes.

It is known that greater MR severity during LVAD support is associated with a reduced likelihood of myocardial recovery (89). In comparison, MR resolution after LVAD was associated with partial or complete myocardial recovery (89, 90). While quantification of MR mainly focuses on hemodynamic parameters and imaging, myocardial biology is expected to have an important impact on MR improvement and myocardial recovery. End stage heart failure itself is well known to demonstrate elevated myocardial inflammatory responses (e.g., innate and adaptive immunity, complement activation) coupled with reduced expression of contractile and energetic/oxidative related proteins (91, 92). We previously reported that increased MR severity is associated with increasing myocardial immune transcriptomic responses (e.g., complement and innate/adaptive immune responses) in patients undergoing LVAD implantation. MR is also associated with decreased expression of transcripts related to structural and proliferative pathways (Figure 3; 91). Consistent with these biological findings, cardiac imaging in patients with degenerative MR show greater myocardial fluorine 18-lebeled fluorodeoxyglucose uptake which reflect increased myocardial inflammation (93). It is recognized that myocardial inflammation with the sequalae of cardiac injury can contribute to worsening of MR (93–95). Sarcoplasmic endoplasmic reticulum Ca^2+^ ATPase 2a (SERCA2a) expression is reduced in the presence of MR and is also associated with worsened LV function and increased ventricular dimensions (96).

Despite the diversity of myocardial molecular signaling underlying the clinical manifestations of MR, this aspect has received relatively little attention for clinical consideration. Published prediction models for LVAD outcomes such as right heart failure and myocardial recovery mainly utilized clinical parameters, imaging, and hemodynamics (28, 29), but not specific biological markers. We previously showed that patients with pre-LVAD moderate-severe MR expressing more myocardial inflammatory transcripts are more likely to resolve their MR (91). Conversely, reduced myocardial inflammation in patients with pre-LVAD moderate-severe MR may indicate a “burnt-out” phenotype with a non-viable and non-contractile LV wall with reduced compliance. These patients are more likely to have persistent MR after LVAD implant due to persistent mitral annular dilatation and poor leaflet coaptation. This is consistent with findings by our group and others that larger LV dimensions represent a more advanced stage of heart failure that is associated with persistent residual MR after LVAD (30, 97). Since severe MR resolves without intervention in about 62–80% of patients, using biomarkers (e.g., inflammation) to identify those likely to have SMR and would benefit from concomitant mitral valve repair can help avoid unnecessary surgical interventions with inherent risks (20, 98).

Return of mitral competence with myocardial recovery during LVAD support may be contributed by reduced myocardial inflammation with the lack of significant MR (89). While inflammatory mediators such as IL-6 and TNFα are known to reduce cardiac function in myocarditis (99, 100). The sustained impact of low simmering degrees of inflammation associated with preoperative and/or residual post-LVAD moderate-severe MR is unknown. However, clinical drug regimens used to promote myocardial recovery in the LVAD setting all have significant anti-inflammatory actions. These agents include mineralocorticoid receptor inhibitors (101), ACE inhibitors or angiotensin receptor blockers (102) and beta-blockers (103). It should be emphasized that correcting the mechanical aspects of mitral regurgitation with valve repair or replacement is also critical. This can restore ventricular geometry, improve contractile mechanics and increase native cardiac ejection (104). Cardiac biology is highly complex in the setting of mechanical circulatory support for end stage heart failure. Molecular biological factors should be incorporated in our prognostic paradigms and therapeutic approaches when managing patients supported durable mechanical devices. It is also likely that novel circulating biomarkers can personalize our approach to targeted surgical heart failure therapies.

## Challenges with evaluating the mitral valve in the left ventricular assist device setting

There are a number of challenges in the study of mitral regurgitation in the LVAD setting, Unfortunately, many studies of the mitral valve in the LVAD setting consists of single institution studies (19, 22, 32, 35, 36) with a low number of patients and thus underpowered. Institutional patient selection also has inherent biases which limit the validity of conclusions. For the relative few multi-institutional studies examining registries (17) and clinical trial data (11), the low data granularity limits our ability to detect patient subsets that may benefit from mitral intervention. Combining the analysis of patients with moderate versus severe MR or not comparing against an appropriate denominator population for example can limit our ability to draw relevant conclusions. Echocardiographic assessment for residual MR is often limited by artifacts from the inflow cannula of the LVAD which makes it difficult to align image windows with the MV (105, 106). Furthermore, the complication of RV failure is often not defined by a quantitative hemodynamic metric which leads to subjectivity. Detailed echocardiographic measurements describing RV function (e.g., tricuspid annular plane systolic excursion, RV ejection fraction, RV dimension) are often not available. Ventricular contractility is also load-dependent and can be temporally variable on echocardiographic examination. RVF was often not defined by a quantitative parameter of RV contractility which contributes to subjectivity. Other concomitant valvular interventions may have also influenced outcomes. Majority of studies are also limited by the retrospective single institutional design with associated biases. Future research protocols would likely benefit from the use of more comprehensive imaging modalities (e.g., 3-dimensional echocardiogram, cardiac computerized tomography or cardiac magnetic resonance imaging) to provide a more detailed assessment of heart function and anatomy before and after LVAD support.

## Conclusion and considerations for future studies

Future studies about MR in the LVAD setting may be designed to consider a number of important issues. Multi-institutional studies enrolling many patients are needed to reveal the impact of significant MR on non-mortality related outcomes in the early to mid-term. The impact of MR on mortality may be better appreciated when improvements in LVAD technology allows longer support durations extending beyond 3 years and/or in those with destination therapy indications. Heart transplantation truncates the duration of LVAD therapy and likely blunts our ability to detect the impact of MR and/or its interventions. Future studies utilizing echocardiography can benefit by incorporating quantitative MR features (e.g., leaflet tethering measurements, quantitative assessment of MR severity, measuring mitral annular diameters, quantifying ventricular morphology), detailed description of hemodynamic parameters with right heart catheterization data (e.g., right heart hemodynamic measurement), and documenting relevant pump settings. Importantly, the duration of LVAD support (e.g., bridge to transplant, destination therapy) will likely determine the impact of residual MR. We have summarized some patient factors that would support concomitant mitral intervention during LVAD implant in Table 1.

**TABLE 1:** Features suggesting concomitant mitral repair for pre-LVAD severe mitral regurgitation should be considered.

Concomitant mitral valve repair may benefit those with the features below
1. Moderate or severe right ventricular dysfunction
2. Moderate or severe tricuspid regurgitation
3. Dilatation of the left ventricle to > 7 cm in diastole
4. Posterior displacement of the mitral coaptation point

Since forward left-sided flow is generally excellent in the presence of a LVAD, the impact of residual significant MR likely rests with increased afterload imposed on the right ventricle. A longer period of exposure would be needed to manifest the negative impact of this on survival and readmissions. Future studies incorporating this interacting variable would be revealing (i.e., duration that the right heart is exposed to significant residual MR). Ultimately, larger studies on this topic including randomized clinical trials will be key. Finally, novel therapies to improve LVAD outcomes (e.g., myocardial recovery) with valvular lesions may incorporate several treatment goals including: (1) reducing wall stress, (2) correction of valve dysfunction to improve hemodynamics, (3) Use of pharmacological therapies that inhibit inflammation, promote cellular (e.g., cardiomyocytes) survival and increase myocardial energy production through activation of beneficial metabolic pathways.

## Author contributions

P-EN, ND, FP, TC, and PT contributed to conception and design of the review. P-EN, MJ, and PT wrote the first draft of the manuscript. ND, MB, and IL wrote the sections of the manuscript. All authors contributed to manuscript revision, read, and approved the submitted version.
